# Sustainability assessment of Construction and Demolition Waste management applied to an Italian case

**DOI:** 10.1016/j.wasman.2021.04.031

**Published:** 2021-06-01

**Authors:** Silvia Iodice, Elena Garbarino, Maria Cerreta, Davide Tonini

**Affiliations:** a European Commission, Joint Research Centre (JRC), Ispra, Italy; b European Commission, Joint Research Centre (JRC), Seville, Spain; c University of Naples Federico II, Department of Architecture, Naples, Italy

**Keywords:** CDW, Life Cycle Assessment, Life Cycle Costing, Land Use, Sustainability Assessment, Selective Demolition, AoP, Area-of-Protection, CAPEX, Capital Expenditures, CLCC, Conventional Life Cycle Costing, CDW, Construction and Demolition Waste, EOLEX, End-of-Life Expenditures, FU, Functional Unit, LCA, Life Cycle Assessment, LCC, Life Cycle Costing, LUC, Land Use Changes, OPEX, Operational Expenditures, RA, Recycled Aggregate, SLCC, Societal Life Cycle Costing

## Abstract

Construction and Demolition Waste represents a priority stream for the European Union and has a large potential for closing the material circulation loop in line with the Circular Economy principles. The present study focuses on the socio-economic and environmental implications of the management of such waste in the Campania Region (Italy), with the aim of documenting the benefits of recycling actions and landfill avoidance. By using local primary data, and complementing them with data from literature and datasets, three scenarios have been investigated: i) *Status Quo*, i.e., a baseline scenario presenting the current management of Construction and Demolition Waste in the Region; ii) a *Linear Economy* scenario, considering the total flow disposed of in landfill and iii) a *Best Practice* scenario based on the implementation of selective demolition practices and increased recycling for the production of high-quality recycled aggregates. Special attention has been paid to the land use and socio-economic implications linked to the management of this flow, which are rarely considered. We quantify that, with the implementation of best practices, ca. 18 Mkg CO_2_ -eq. can be saved annually relative to the *Status Quo* alongside creating additional 1,000 jobs-eq. and incurring important benefits on land use. The results stress that the potential environmental and social benefits of selective demolition and best practices are significant, but the incurred economic costs may hinder their application and the resulting development of more circular economy actions in the construction sector, highlighting the need for incentives and tools to facilitate this transition.

## Highlights

• We performed a full sustainability assessment with primary regional data.
• Best practices with selective demolition generate higher conventional costs overall.
• Best practices with selective demolition generate environmental and social benefits.
• Costs for best practices are mainly incurred by the selective demolition phase.
• Instruments appear needed to compensate increased costs and facilitate transition.

## 1 Introduction

Construction and Demolition Waste (CDW) is a significant waste stream in Europe in terms of quantities generated and recyclability potential. CDW accounts for about 25–30% of the total waste generated in the EU (European Commission, 2019a) and it is therefore considered a priority waste stream also in view of the impacts caused by its mismanagement (Duan et al., 2015, Marzouk and Azab, 2014). The European Commission has recently developed the EU Construction and Demolition Waste Protocol and Guidelines, proposing improvements in waste identification, source separation and collection, logistics, processing and quality management (Commission, 2016, Commission, 2018). On the same lines, Directive 2008/98/EC establishes a target to prepare for re-use, recycle or recover a minimum of 70% (by weight) of non-hazardous CDW by 2020. According to its amendment in 2018, this Directive considers the setting of new targets for CDW and its material-specific fractions by 2024. Moreover, in the framework of the European Green Deal (European Commission, 2019b), the new Circular Economy Action Plan considers CDW as a priority stream for closing the material loop thanks to its potential to produce secondary raw materials (European Commission, 2020a), and to the ‘Renovation Wave’ goals (European Commission, 2020b). Against these ambitions, as for today considerable efforts are however still necessary to fulfil such goals (Kabirifar et al., 2020, López Ruiz et al., 2020). Looking at the Italian context, CDW is classified as ‘special waste’ (Legislative Decree 152/2006) i.e., waste deriving from productive activities that is managed and disposed of by authorized companies. The most recent reports on special waste (Ispra, 2019, Ispra, 2020) estimate an annual production of about 40 Mt in the country. Specifically, in the Region Campania, focus of the present study, about 2.9 Mt are generated annually representing ca 40% of the total generated waste in the Region. In the past, landfilling was a usual practice when dealing with inert waste (Blengini and Garbarino, 2010), but recently, alternative options have been explored to valorise this stream. Notably, CDW can be turned into secondary raw materials known as Recycled Aggregates (RAs) following appropriate recycling processes (Blengini and Garbarino, 2010; Badino et al., 2007; Borghi et al., 2018; Silvaet al., 2014). To this aim, they need to follow construction product standards (such as EN 12,620 Aggregates for concrete or EN 13,242 – Aggregates for unbound and hydraulically bound materials for use in civil engineering work and road) and some specific requirements (e.g. attachments to the Italian Ministerial Communication n. 5205/2005). According to Blengini and Garbarino (2010) and Borghi et al. (2018), and greatly in line with the above-mentioned Communication, the following main categories of RAs have been identified: i) type A: considered as a high-quality material, with structural properties able for concrete production and road foundations; ii) type B: a medium quality material used for road, airport and harbour construction as well as unbound material in the embankment body, in sub-base layers and in layers with anti-freezing, anti-capillary and drainage properties; iii) type C: a lower quality material, used for environmental fillings and rehabilitation of depleted quarries and landfill sites.

Many studies investigated the impacts related to CDW management strategies. In most of the cases, the Life Cycle Assessment (LCA) methodology has been used. This happens in the study by Li et al. (2020) and Mercante et al. (2012), where LCA is used to assess the environmental impacts of CDW in specific case studies, or in the evaluation performed by Butera et al. (2015) aimed at comparing the best possible use of secondary raw materials. Yazdanbakhsh (2018) proposed a LCA framework for modelling alternative waste management approaches, while Coelho and de Brito (2010) applied the LCA to the whole life cycle of a building, beyond the waste management phase. Most of these studies focused on the production of Recycled Aggregates (RAs) (Zhang et al., 2020). In this context, it is worth mentioning the analysis performed by Blengini and Garbarino (2010), who developed a combined Geographic Information System and LCA model to understand the potentialities of RAs helping the transition towards a sustainable supply mix of natural and recycled resources. (Borghi et al., 2018) focused on the relationship between mixed non-hazardous CDW and the production of medium and low-quality RAs, to understand how to improve the production of high-quality secondary raw materials. The authors provided general recommendations to enhance the performance of the system from an environmental point of view.

All in all, reviewing the available literature, we observed that very few studies focused on the socio-economic and land use impacts of CDW management, especially in relation to selective demolition and best practices. Only Pantini and Rigamonti (2020), that we are aware of, analysed and compared with LCA selective demolition versus traditional, showing how the benefits are strictly linked to the characteristics of buildings and the RAs local market. Wang et al. (2019), while dealing with the introduction of a waste management fee, introduced the possibility of involving the civil society in decision-making. Both studies only address the environmental issues, and a multidimensional perspective is missing. As for the land use impacts, the only study we found is the one of Bidstrup et al. (2015), who proposed an integration between LCA and Strategic Environmental Assessment. The authors quantified LUC and showed the usefulness of LCA for spatial planning, though without evaluating economic and social impacts by means of tools such as Conventional Life Cycle Costing (CLCC) or Societal Life Cycle Costing (SLCC). Economic costs were instead evaluated in Di Maria et al. (2018), where a combined LCA and LCC methodology is used to assess the environmental and economic implications of four different CDW scenarios and Di Maria et al. (2020) where, through a combination of economic and environmental results, policy makers are supported in the implementation of new CDW recycling opportunities. Yet, a zoom-in on land use and social aspects is missing.

**Fig. 1:**
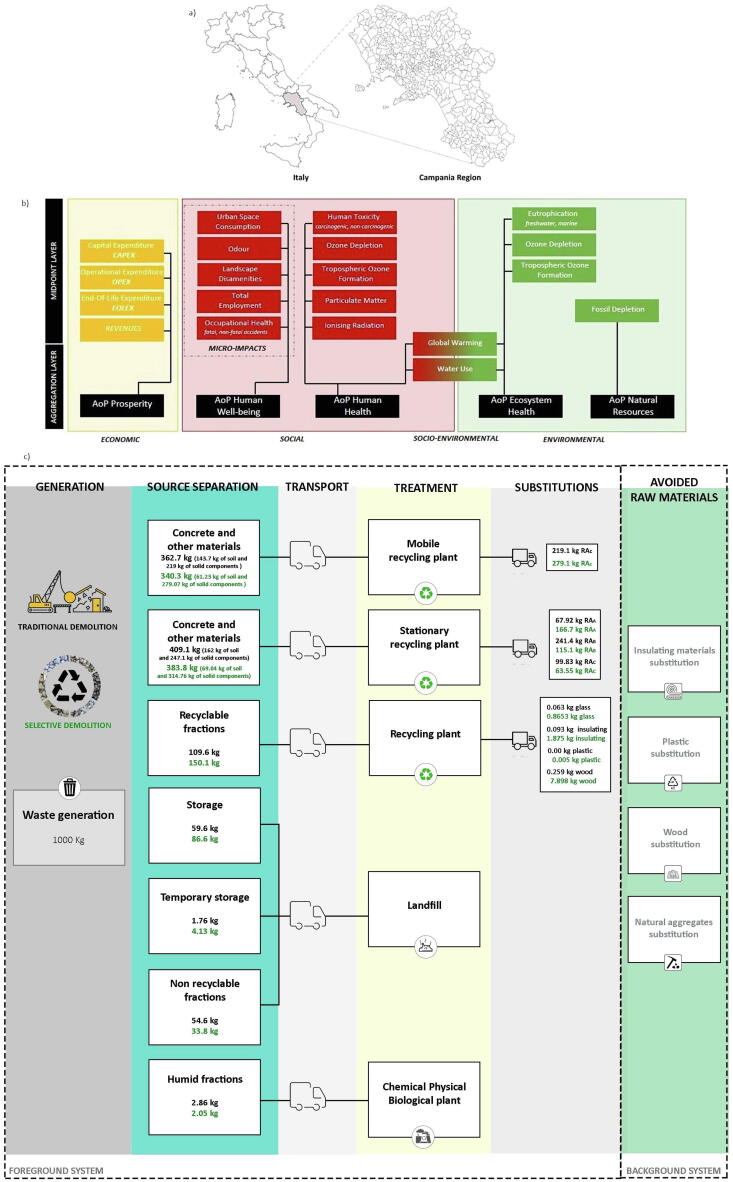
Illustration of a) Campania Region (Italy; focus area of the assessment), b) the sustainability framework as applied in the study (20 multidimensional indicators covering 5 Areas-of-Protection at the end-point level, based on Taelman et al., 2020). AoP: Area-of-Protection, c) CDW flows distribution in the scenario Status Quo (black), where traditional demolition is applied, and Best Practices (green), where selective demolition and increased recycling are assumed to be applied. RA: Recycling Aggregates. (For interpretation of the references to colour in this figure legend, the reader is referred to the web version of this article.)

Having this in mind, the current study is aimed at presenting the results of a sustainability assessment of the CDW management in the Campania Region (Italy) (Fig. 1a). This assessment has been carried out by using a multidimensional sustainability framework, which covers the socio-economic and environmental pillars as defined in Taelman et al. (2020) (Fig. 1b) and is based on local stakeholder priorities. We further complemented the analysis with a SLCC including externality costs and land use impacts in order to show a broader picture of the CDW management consequences. To this aim: i) we collected local primary data on CDW composition, flows and treatment technologies in terms of input–output data on material flows, energy use and emissions. These primary data have been complemented with Ecoinvent 3.6 datasets and recent literature sources; ii) we designed and compared three scenarios to recommend sustainable solutions for the local management of CDW, which are widely applicable beyond this local context; iii) we strengthened the assessment through an uncertainty propagation analysis, based on recent developments in the field (Bisinella et al., 2016).

## 2 Materials and methods

### 2.1 Case study

The focus area selected for the analysis is represented by the Campania Region, Italy (Fig. 1a; 5.8 million inhabitants; 13,671 km^2^; 2.9 Mt/year of CDW generated). CDW is managed in the Campania Region following the indications and objectives of the Regional Plan for the management of special waste (2012). Some of these indications have been addressed in the present analysis, particularly to what concerns ensuring the self-sufficiency and the environmental and economic sustainability of an integrated and coordinated management system, by minimizing its impact on human health and the environment alongside the social and economic costs.

### 2.2 Scope and functional unit

The goal of the study is to evaluate the sustainability of a few scenarios for CDW management. The Functional Unit (FU) is the management of 1 t of CDW generated in the Campania Region (Italy), where the annual generation equals 2.9 Mt (wet weight) (Ispra, 2017, Ispra, 2018). CDW is generated along the life cycle of a building or infrastructure, including construction and demolition activities, micro renovations carried out independently or other activities dealing with construction materials. The material fraction composition of CDW depends on the applied demolition practices (see section 3.1). We consider current technologies and the Italian energy system as expected for the period 2020–2030 (Keramidas et al., 2021).

The assessment was facilitated with the software EASETECH (Clavreul et al., 2014) and a consequential approach was applied. This approach is aimed at describing the effects of changes within the life cycle (Ekvall, 2002) due to modifications of the life-cycle inventory determining chains of cause-effect relationships (Curran et al., 2005, Zamagni et al., 2012).

### 2.3 Sustainability assessment framework

The assessment applies the sustainability framework developed in Taelman et al. (2020), encompassing five areas-of-protection (AoPs) at the endpoint level and selecting a total of 20 different environmental, social, and economic indicators at midpoint level among those originally established in the framework (Fig. 1b). The framework also proposes a final ranking of the scenarios at the AoPs level, through the application of the ELECTRE II Multi-Criteria method (Figueira et al., 2005) after min–max normalisation (rescaling of the characterised results) and weighting of the impact categories following stakeholder's priorities. This approach was chosen instead of applying normalisation references and weights to derive a single score because of the lack of normalisation references for most of the socio-economic indicators. We report hereafter a brief description of the socio-economic indicators only. For more details on how this framework was built, on the two-stages participatory process set up for selecting the indicators and on the model developed for the MCDA application, the reader is referred to Taelman et al. (2020) and Tonini et al. (2018).

#### 2.3.1 Human well-being (social) indicators

Disamenities, reflecting an unpleasant character, comprise visual impacts and other negative aspects due to the presence of waste treatment plants, such as noise and odours. In general, they are calculated through the Hedonic Price Method (HPM) (Rosen, 1974), which assesses how and to what extent a certain factor can affect market prices of buildings. It is indeed demonstrated that the house price decreases as the distance between the house and the disamenity increases, reaching zero at a defined distance. The application of this methodology allows to include in the sustainability framework local disamenities-related impacts, considering the variation in property prices at different distance ranges (0–1 Km, 1–2 Km, 2–3 Km, 3–4 Km, 4–5 Km) until a maximum distance beyond which WM facilities do not affect market prices anymore. If it is possible to obtain information on the market values as well as on some characteristics of the properties (for example: size, typology and people living), it can be applied a linear regression, as proposed by Rivas Casado et al. (2017). In the absence of information on the characteristics of housing, a simplified formula proposed by European Commission (2014) can be applied. According to European Commission (2014), the result is a negative impact represented by a fixed amount that does not change with the amount of waste that is disposed of or treated. Therefore, disamenity costs represent a fixed externality, but it can be represented as a cost per tonne of waste for a specific necessity such as that of comparing different scenarios. Details on the calculation of the ‘Landscape Disamenities’ indicator for the present case study are provided in Tables S6a-S6b (SI). Alongside Landscape Disamenities, another significant micro impact affecting ‘Human Well-Being’ is represented by ‘Odour’, in terms of odour footprint calculated using linear midpoint characterization factors based on the potential malodorous air generated by the compound released to the atmosphere.

‘Occupational Health’ indicator is related to the assessment of working conditions with reference to safety and it is calculated using the ratio between the total labour and a place-based accidents rate that includes fatal and non-fatal accidents (Table S7, SI). Accidents rates in the waste management sector (including a distinction between accidents involving or not a means of transportation) are retrieved from the open data available from the National Institute for insurance against accidents at work and data on the number of employees in the waste management sector are retrieved from the open data available from the National Institute of Statistics. Further details are available in SI, section 7.

The local indicator ‘Total Employment’ refers to the number of jobs created in the foreground system with reference to the assessed activity. The indicator is calculated through the ratio between the number of employees and the FU. As regards ‘Urban Space Consumption’ indicator, it considers the urban space used by the Waste Management facilities compared to the total surface of the Region under study. In the case of ‘Private Space Consumption’, this indicator has not been included in the analysis of CDW, since in general the management of this flow does not involve the use of private spaces.

#### 2.3.2 Prosperity (economic) indicators: CAPEX, OPEX, EOLEX, REVENUES

OPEX refers to the operational costs linked to the CDW management (€/FU), CAPEX assesses the total costs to acquire, maintain or upgrade the physical assets of a waste management system (€/FU), EOLEX considers the costs to dismantle facilities (€/FU) and REVENUES considers the revenues coming from the sales of products. For the specific description of the assessed indicators, see the SI of this paper or of Taelman et al. (2020).

### 2.4 Scenarios assessed

The analysed waste streams comprise direct flows, directly sent to treatment plants, and secondary ones, which are related to the intermediate management operations in the temporary storage facilities before landfilling (Fig. 1c).

To support the decision-making for CDW management and Eco-Innovative Solutions selection (Garzilli et al., 2020), we designed and assessed three scenarios relevant to the focus area: first, a *Linear Economy* scenario to illustrate the benefits of avoiding landfilling, based on the hypothesis of entirely disposing of the CDW flow in landfill. Second, we considered the environmental, social, and economic impacts associated with the current CDW management in the Region (*Status Quo* scenario), based on the implementation of the following plants:

i) Stationary recycling plants, which treat the CDW inert fractions for producing RAs. These plants usually show a higher level of technology than the mobile ones, including sorting equipment for the separation of unwanted fractions (Blengini and Garbarino, 2010). Stationary plants produce high, medium and low-quality RAs (type A, B and C, respectively). Through a survey to the main stationary plants located in the Campania Region, the rate of production of the three RA types has been estimated to 17% for high-quality RAs (type A), 59% for medium-quality RAs (type B) and 24% for low-quality RAs (type C). Details on the respondents are provided in Table S3 (SI).

ii) Mobile recycling plants usually treat smaller CDW quantities in temporary demolition worksites and use basic technologies. Mobile plants typically produce low-quality RAs (type C) (Blengini and Garbarino, 2010).

iii) Recycling plants that treat the other recyclable fractions in the CDW stream (such as plastic, metals, glass, wood and insulation materials).

iv) Landfills for disposing of the non-recyclable CDW fraction, which are mainly located outside the Region.

v) A minor flow sent to Chemical-Physical-Biological plants for treating the small humid fraction, for which only transport was modelled. While emissions and leachate generation can be considered negligible, we accounted for the emissions coming from the consumption of energy, land use and infrastructure (Penteado and Rosado, 2015).

Third, we developed a *Best Practice* scenario to quantify the potential savings achieved applying advanced demolition and treatment techniques, to the aim of increasing material recovery and recycling. This scenario is based on the following assumptions:

i) Systematic application of selective demolition, also known as ‘construction in reverse’ or ‘deconstruction’ (Pantini and Rigamonti, 2020). This type of demolition remains rarely applied but holds a great potential. The difference with the traditional demolition lies in the separation of waste at the place of generation thanks to a sequence of demolition activities, which allows separating and sorting building components and materials (Pantini and Rigamonti, 2020). This increases the capture rate and quality of waste (i.e. overall recyclability). Details on the economic and environmental inventory used for modelling selective and traditional demolition are available in Table S4e of the SI.

ii) Increased production of high-quality RAs (type A), which can be used in low-strength structural concrete. This is considered an important contribution to close construction materials cycles, as it decreases the amount of residual CDW to be managed, increases the economic value of the recycled component and reduces the quantity of Natural Aggregates (NAs) used, as also explained in Di Maria et al. (2018) and Penteado and Rosado (2015).

### 2.5 System boundary

The system boundary includes all the activities in the waste management life cycle, i.e. collection, transport, treatment, transportation of treated residues and/or products to end-use or further applications, and possible final landfilling. Furthermore, the secondary raw materials generated through the CDW recycling are credited through system expansion, by substituting the correspondent market products. Particularly, the modelling strives to account for the impacts related to the substitution of NAs from quarrying activities with RAs, in order to quantify the avoided social, economic and environmental impacts. This can ultimately lead to a gradual reduction of the extractive activities. This way, the impacts related to the production of RAs are compared to the ones related to the production of NAs from limestone quarries in the Campania Region to account for the avoided extraction of raw materials. For the substitution of NAs with RAs, replacement coefficients considering the quality of RAs and their market demand (as proposed by Borghi et al., 2018) have been applied:

R = Q1 * Q2 * M

Where Q1 is the quality in terms of purity of the flow, while Q2 is linked to the technical characteristics of RAs compared to those of the substituted material. Finally, M is the market coefficient, i.e. the ratio between the amount of RAs sold and produced in a recycling plant in a defined period. M usually varies between 0 and 1, according to the market attractiveness. For the present case study, it is set equal to 1, assuming a time horizon in which all the produced RAs are used and satisfy the market demand. The replacement coefficients resulted equal to 1, 0.97 and 0.86, for RAs of type A, B, and C accordingly. Other important substitutions occur between recycled plastic, glass, wood, and insulation materials and the corresponding raw materials available on the market. We excluded metal components from the analysis, assuming that they are typically separated at source and sent to recycling. Indeed, a large amount of metals is selectively separated during demolition by means of mechanical equipment and does not enter the CDW flow to be further treated. As far as the material substitutions are concerned, sorting and recycling efficiencies developed in Pantini and Rigamonti (2020) and Faraca et al. (2019) have been adopted (sorting rates were 47% for wood, 72% for insulation and plastic and 57% for glass respectively).

### 2.6 Uncertainty analysis

An uncertainty analysis based on the propagation of the parameter uncertainty on the results through a Montecarlo simulation has been applied, using a triangular distribution to represent the uncertainty range as suggested in Bisinella et al. (2016). A range of ± 20% around the default value has been assumed for most of the parameters (around 60% of total parameters). For the remaining parameters, specific variations have been identified using local data, literature or guess-estimates. In particular, transport parameters have been varied according to the maximum distance that can be travelled from the Campania Region to the most distant Italian Regions in which CDW is treated. Finally, for the most uncertain parameters, for which it was not possible to obtain primary data (especially as regards the economic parameters in terms of Labour, OPEX, CAPEX and REVENUES), a wider variation oscillating between ± 50% and ± 100% was applied. CAPEX of stationary recycling plants and landfills are affected by the land acquisition value, which varies according to the land use on which the plant is installed (between 2 €/m^2^ for agricultural land, used as minimum value for the uncertainty, and 150 €/m^2^ for commercial land, established as maximum value); for landfill the agricultural land value is assumed. For mobile recycling plants, OPEX includes the land rent value, for which the minimum and maximum values are established through a market research (3,600–7,848 €/year). Further information is available in the SI (section 4).

## 3 Inventory data

To describe the foreground system, we used official primary data provided by the environmental regional agency of the Campania Region (reference year: 2015). This included local primary data on: i) CDW composition, ii) CDW flows (i.e. share to landfilling, recycling, etc.; Table S2, SI), iii) energy, electricity, material, fuels and resource provision, iv) local data on accidents rates (Table S7, SI), land occupation of facilities, quarries and containers (Table S5a of the SI). Complementary background data for modelling waste treatment technologies (e.g. input–output data related to material, energy use and emissions) were taken from Ecoinvent centre 3.6 (Ecoinvent centre, 2019). Transport distances were calculated as a weighted sum from the centroid of the Campania Region to the centroid of the receiving Italian Regions, also by using a Geographic Information System.

### 3.1 Material composition, flows and quantity

The material fraction composition of CDW currently reported by ARPAC and Ispra (2017) refers to flows originating from traditional demolition practices (see Section 3.2). This composition was derived from primary data and aggregated according to macro-categories. For this study, we also estimated the material flows that would potentially originate from selective demolition practices. The chemical composition has been approximated with selected material fractions from the EASETECH database (Butera et al., 2015, Clavreul et al., 2014).

### 3.2 Traditional versus selective demolition practices: Material flows

**Fig. 2:**
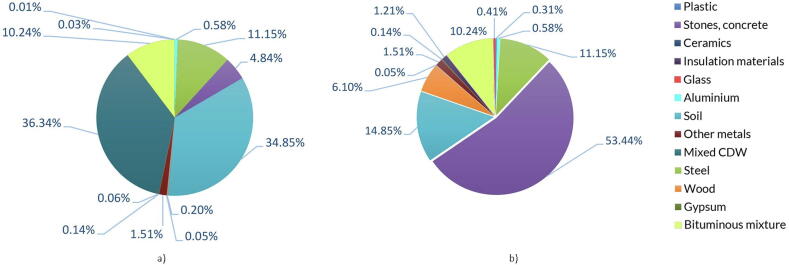
Illustration of a) Material composition of the *Status Quo*, b) Material composition of the *Best Practice* scenario.

The CDW streams generated with the current demolition practices is detailed in Fig. 2a and Table S1a (SI), quantified based on the NACE 41, 42, 43 codes elaboration, also considering the possibility that economic activities, other than the construction ones, can produce CDW, and is based on the elaboration of the European Waste Catalogue (EWC) code 17.

A new composition has been estimated using literature references, in particular the elaboration from Metabolic (2020) and Lavagna et al. (2018) that specify the material content breakdown for buildings to define the potential recovery rate of each fraction when applying selective demolition. Lavagna et al. (2018) consider 24 different dwelling archetypes selected to be as much representative as possible of the existing building types in Europe, while the analysis performed by Metabolic (2020) focus on residential and commercial buildings in The Netherlands. Following these references, a new composition is detailed in Fig. 2a and Table S1b (SI). Notably, it is possible to observe the increase in the share of the stones and concrete fraction, which then allows an increase in the production of high-quality RAs. The max recovery of the stones and concrete fraction via selective demolition is estimated up to ca. 53%, allowing an increased production of high-quality RAs from ca. 17% (traditional demolition) to ca. 53%. See additional details on material fractions in SI, section 1. Other significant changes refer to the composition of plastic, insulation materials, wood, and glass.

Selective demolition also implies different electricity and diesel consumptions, as well as different labour costs. We estimated energy consumptions and labour costs based on Pantini and Rigamonti (2020) and Coelho and De Brito (2013). The electricity and diesel consumptions for traditional demolition were 0.1 kWh/t and 1.8 MJ/t, respectively, against 1.6 kWh/t and 2.8 MJ/t for selective demolition. The labour costs were estimated based on the study performed by Coelho and De Brito (2010)) as 1.2 €/t and 6.2 €/t for traditional and selective demolition, respectively. The authors in their study considered townhouses of 100 m^2^ and similar patterns are also confirmed by other studies, which consider different construction typologies (Dantata et al, 2005) or different databases (Vázquez-López et al., 2020).

### 3.3 Land use and land use change (LUC)

Land use refers to the human activities carried out in a certain land cover. Therefore, it concerns the functional dimension and the socio-economic activities that characterise a certain area.

Land use is divided into two categories: ‘land occupation’ and ‘land transformation’. The first one refers to the continuous use of an area for a certain human activity, while the second one represents a change in use or management of soil caused by human action, referring to the change from one category of land use to another. In this regard, direct land use change (dLUC) represents the transformation caused directly by the expansion of a certain land use activity and indirect land use change (iLUC) represents the transformation caused indirectly from the competitive use of the land, beyond the borders of the system studied and which is attributable to the system studied. For each considered treatment plants, the evolution of land use in relation to the life cycle of soil was analysed, considering land transformations and land occupations. Furthermore, site-specific land- use and land use-change data have been collected in relation to the limestone quarries in the Campania Region (SI, Section 5).

### 3.4 Waste technologies and costs

The life cycle inventory for each waste treatment technology was compiled based on information from various sources (SI, section 2). With respect to the calculation of the annualised CAPEX (capital expenditures expressed as cost per tonne of feedstock treated), OPEX (operational expenditures, as cost per tonne of feedstock treated) and externalities (environmental price of the emissions),we conformed to the calculation method widely used in recent studies focusing on waste economics (Hunkeler, 2008, Martinez-Sanchez et al., 2015; Martinez-Sanchez et al., 2016). To represent externalities, the shadow prices of the environmental emissions as reported in de Bruyn et al. (2017) were used. The revenues were derived based on the current market prices for recycled material, e.g. recycled aggregates. Last, the total employment was derived knowing the labour input to the individual processes or technologies throughout the foreground system assessed.

## 4 Results

**Fig. 3:**
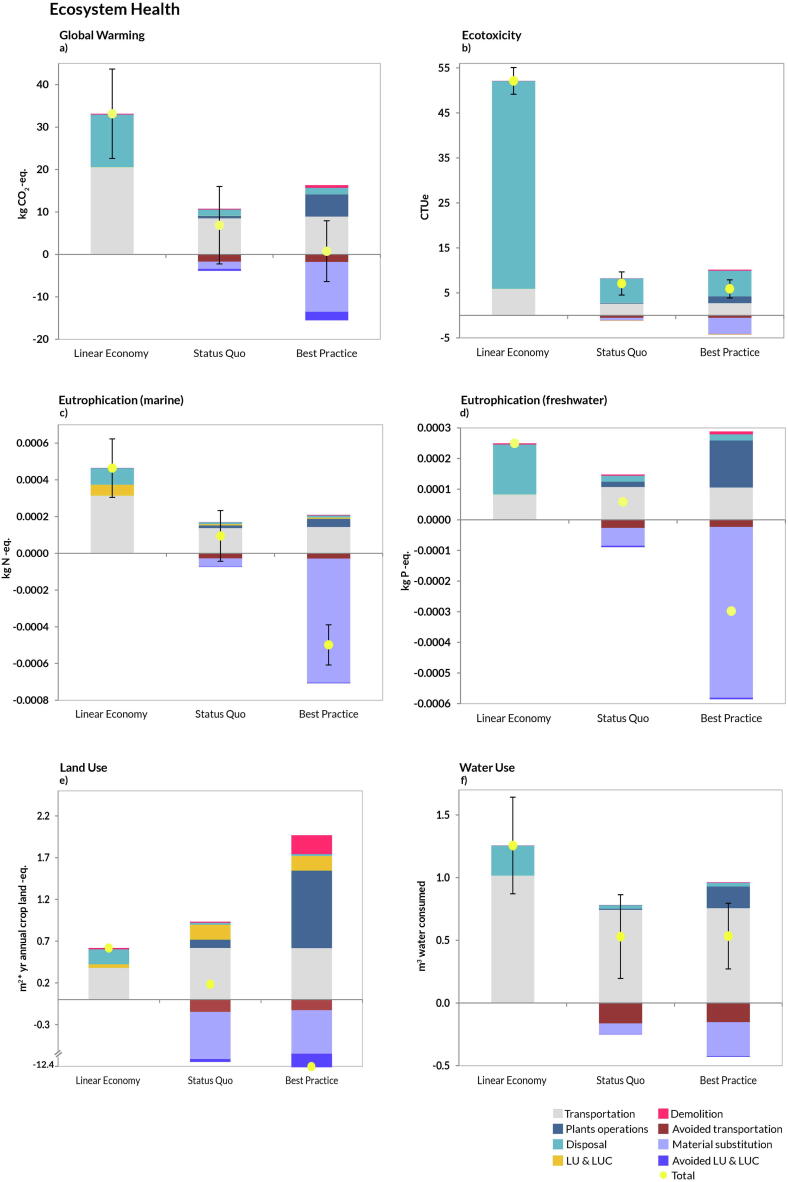
Disaggregated life-cycle results for the Area-of-Protection ecosystem health. (FU: management of 1 t CDW). Positive values indicate burdens, while negative indicate savings. Plants operations: all activities involving material recycling (aggregates, wood, plastic, etc.); Material substitution: credits from replacement of virgin materials; Transportation: impacts related to transport of CDW flow to the management facilities and transport of RAs to the construction sites; Avoided transportation: saving related to avoided NAs transportation from quarry sites to plant facilities; LUCs induced: impacts due to land occupation, land transformation and land use changes (both direct and indirect); LUCs avoided: avoided land occupation, transformation and land use changes due to the reduction of mining activities from quarries; Disposal: all the impacts incurred by landfilling operations; Demolition: impacts coming from the process of demolition (both traditional and selective). Total represents the sum of all contributions. The uncertainty bar represents plus minus one standard deviation.

The breakdown of the midpoint impacts is displayed for the 20 indicators grouped according to five individual AoPs (Fig. 3-to-7). Herein the key differences between the *Linear Economy*, B*est Practice* and S*tatus Quo* scenarios are outlined, whereas for a detailed description of the results the reader is referred to section 4.1-to-4.3. Overall, in the *Status Quo* the CDW management resulted in a net impact in most of the environmental categories, i.e. the savings determined by recycling operations were not enough to compensate for the burdens incurred by management and treatment processes. Yet, this scenario performed better than the *Linear Economy*, used here for benchmarking and quantifying the benefits arising from avoiding landfilling. Relative to both, the *Best Practice* scenario showed overall lower impacts and/or net savings albeit this was not the case in the AoP Prosperity, where higher total costs (sum of OPEX, CAPEX, EOLEX, and REVENUES) were incurred notwithstanding the increased revenues generated from material recycling. With respect to the breakdown of the individual impact contributions, CDW treatment and transport originated the main burdens, while the most important contributions to the savings came from the substitution of conventional market products and associated processing (the recyclable materials and the avoided extraction of raw materials from quarries), avoided transport and LUCs.

### 4.1 Environmental and resource-related impacts: Ecosystem Health, Human Health, and Natural Resources

In the AoP Ecosystem Health, the *Best Practice* scenario performed best in all categories except for Water Use and the *Status Quo* was always better than the *Linear Economy* (Fig. 3). This was mainly due to the increased savings from material substitution thanks to the recycling operations. It should be noticed that transportation was also a major contribution to the burdens especially for Global Warming (Fig. 3a), Eutrophication (both marine and freshwater; Fig. 3c,d) and Water Use (Fig. 3f), mainly owing to the emissions of CO_2_ and NO_x_, and to water consumptions for diesel–fuel production. While LUCs were accounted for across all categories, they resulted a non-negligible contribution only for the Land Use category (Fig. 3e).

**Fig. 4:**
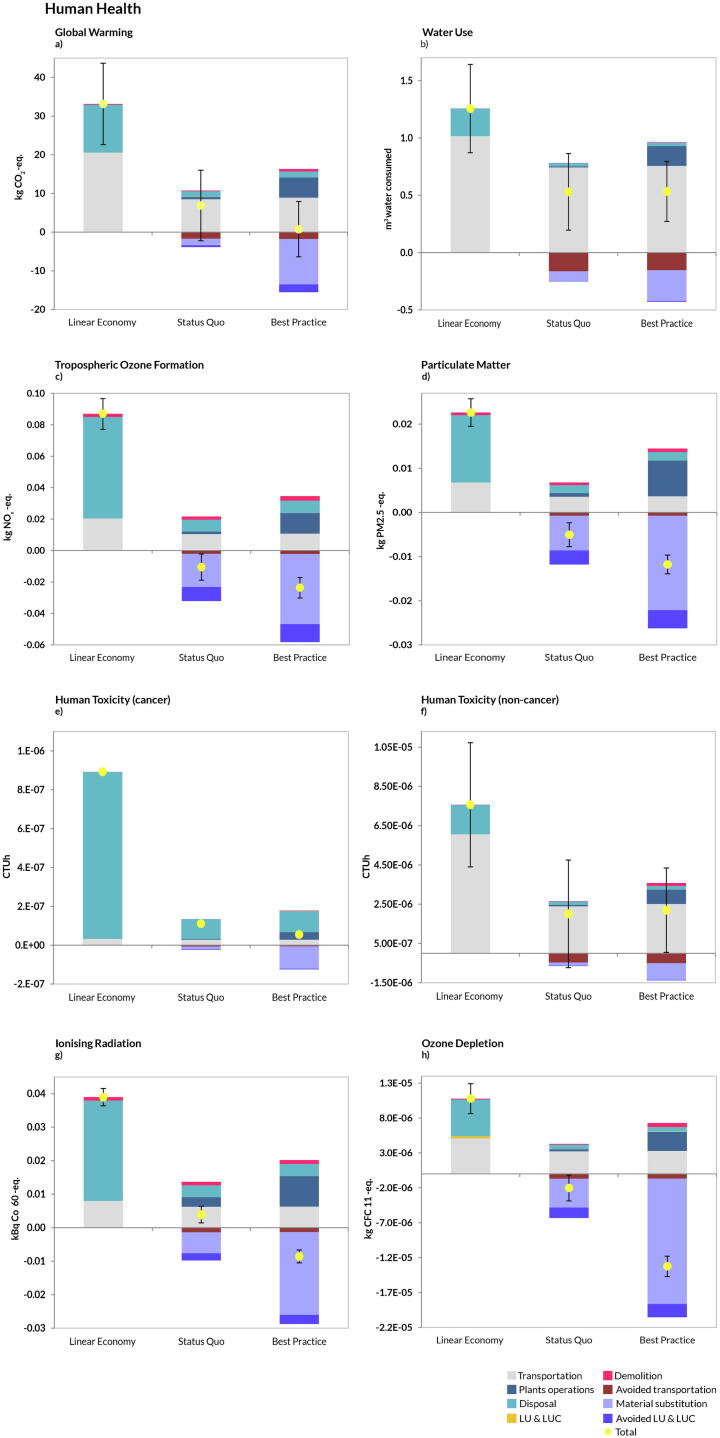
Disaggregated life-cycle results for the Area-of-Protection human health. (FU: management of 1 t CDW). Positive values indicate burdens, while negative indicate savings. Plants operations: all activities involving material recycling (aggregates, wood, plastic, etc.); Material substitution: credits from replacement of virgin materials; Transportation: impacts related to transport of CDW flow to the management facilities and transport of RAs to the construction sites; Avoided transportation: saving related to avoided NAs transportation from quarry sites to plant facilities; LUCs induced: impacts due to land occupation, land transformation and land use changes (both direct and indirect); LUCs avoided: avoided land occupation, transformation and land use changes due to the reduction of mining activities from quarries; Disposal: all the impacts incurred by landfilling operations; Demolition: impacts coming from the process of demolition (both traditional and selective). Total represents the sum of all contributions. The uncertainty bar represents plus minus one standard deviation.

**Fig. 5:**
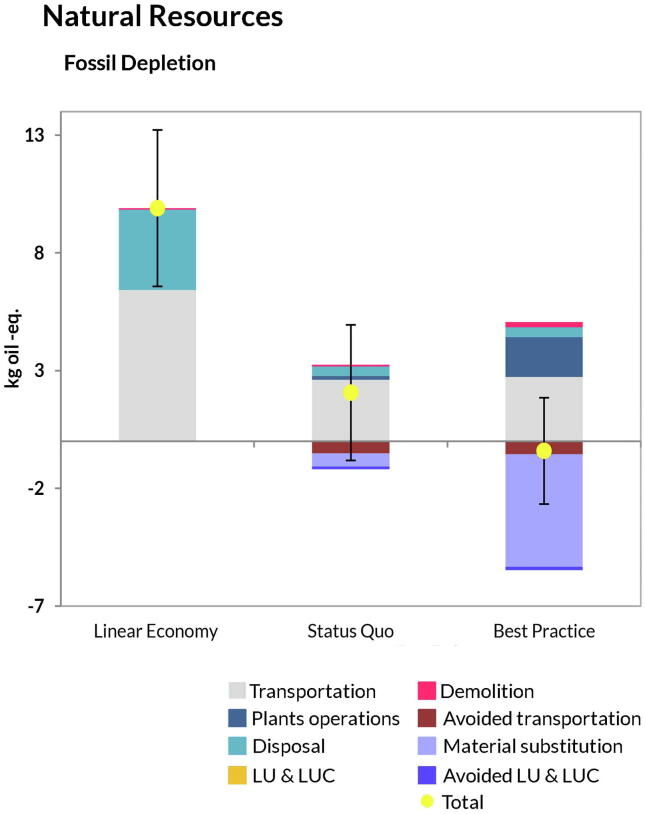
Disaggregated life-cycle results for the Area-of-Protection natural resources. (FU: management of 1 t CDW). Positive values indicate burdens, while negative indicate savings. Plants operations: all activities involving material recycling (aggregates, wood, plastic, etc.); Material substitution: credits from replacement of virgin materials; Transportation: impacts related to transport of CDW flow to the management facilities and transport of RAs to the construction sites; Avoided transportation: saving related to avoided NAs transportation from quarry sites to plant facilities; LUCs induced: impacts due to land occupation, land transformation and land use changes (both direct and indirect); LUCs avoided: avoided land occupation, transformation and land use changes due to the reduction of mining activities from quarries; Disposal: all the impacts incurred by landfilling operations; Demolition: impacts coming from the process of demolition (both traditional and selective). Total represents the sum of all contributions. The uncertainty bar represents plus minus one standard deviation.

A similar pattern was observed for the AoP Human Health (Fig. 4). While in the energy-related categories the scenario *Best Practice* was always preferred because of increased savings from material substitution, reduced transportation and LUCs owing to increased recycling (Fig. 4a-to-h), the savings in the toxicity categories (Fig. 4e,f) were mainly related to reduced disposal of materials in landfill and avoided transportation of natural aggregates. This was also the case for the AoP Natural Resources, where increased recycling and substitution of virgin material compensated for the fuel and energy expenses incurred by the technologies involved in the CDW treatment (Fig. 5). As for the uncertainty bar, it can be observed that the scenarios *Status Quo* and *Best Practice* largely overlapped in Global Warming, Water Use and Fossil Depletion.

### 4.2 Social impacts: Human Well-being

**Fig. 6:**
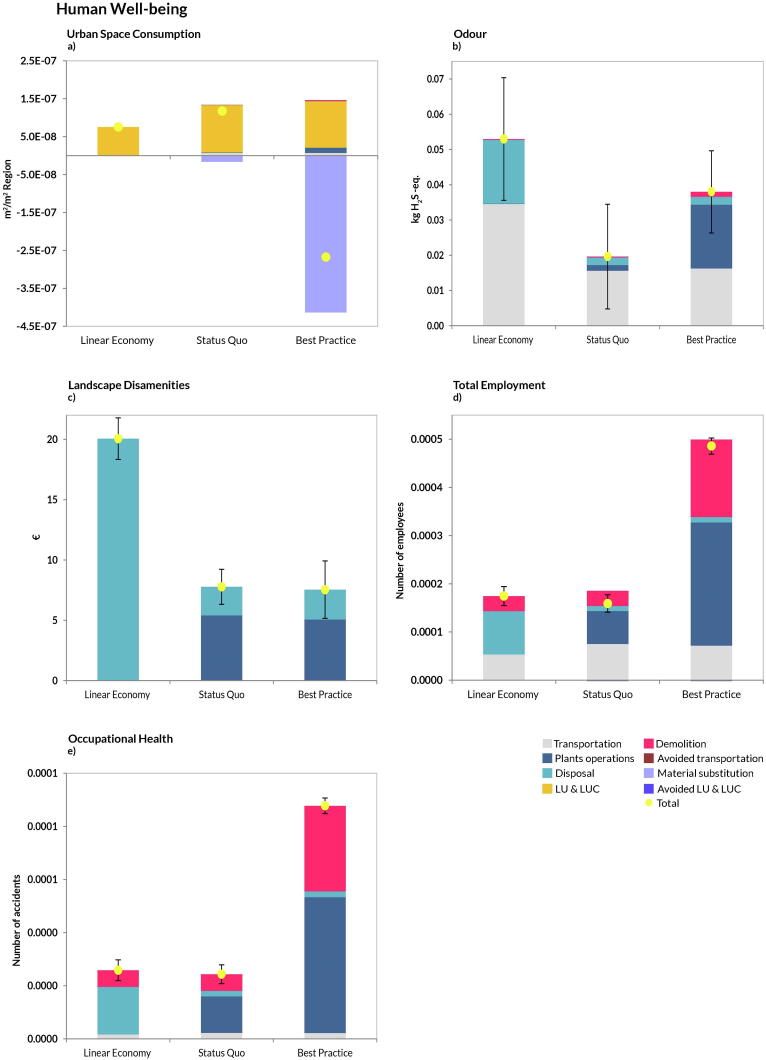
Disaggregated life-cycle results for the Area-of-Protection human well-being (FU: management of 1 t CDW). Positive values indicate burdens, while negative indicate savings. Plants operations: all activities involving material recycling (aggregates, wood, plastic, etc.); Material substitution: credits from replacement of virgin materials; Transportation: impacts related to transport of CDW flow to the management facilities and transport of RAs to the construction sites; Avoided transportation: saving related to avoided NAs transportation from quarry sites to plant facilities; LUCs induced: impacts due to land occupation, land transformation and land use changes (both direct and indirect); LUCs avoided: avoided land occupation, transformation and land use changes due to the reduction of mining activities from quarries; Disposal: all the impacts incurred by landfilling operations; Demolition: impacts coming from the process of demolition (both traditional and selective). Total represents the sum of all contributions. The uncertainty bar represents plus minus one standard deviation.

In the AoP Human Well-being, the B*est Practice* scenario performed best in all categories except for Odour (Fig. 6b) and Occupational Health (Fig. 6e), while for Landscape Disamenities, the performance was comparable to the *Status Quo*. The *Status Quo* was always better than the *Linear Economy* (Fig. 6), except for the category Urban Space Consumption (Fig. 6a). Notably, the *Best Practice* scenario achieved the best score in Total Employment (Fig. 6d) and in the Urban Space Consumption category, thanks to the increased number of treatments/operations involved (mainly recycling) and to the avoided land use impacts connected to such activities. It should be noticed that transportation was again a major contribution to the burdens, especially for the category Odour (Fig. 6b) and Total Employment (Fig. 6d). As for the uncertainties, the *Status Quo* and the *Linear Economy* scenarios largely overlapped in the categories represented by Odour and Landscape Disamenities.

### 4.3 Economic impacts: Prosperity

**Fig. 7:**
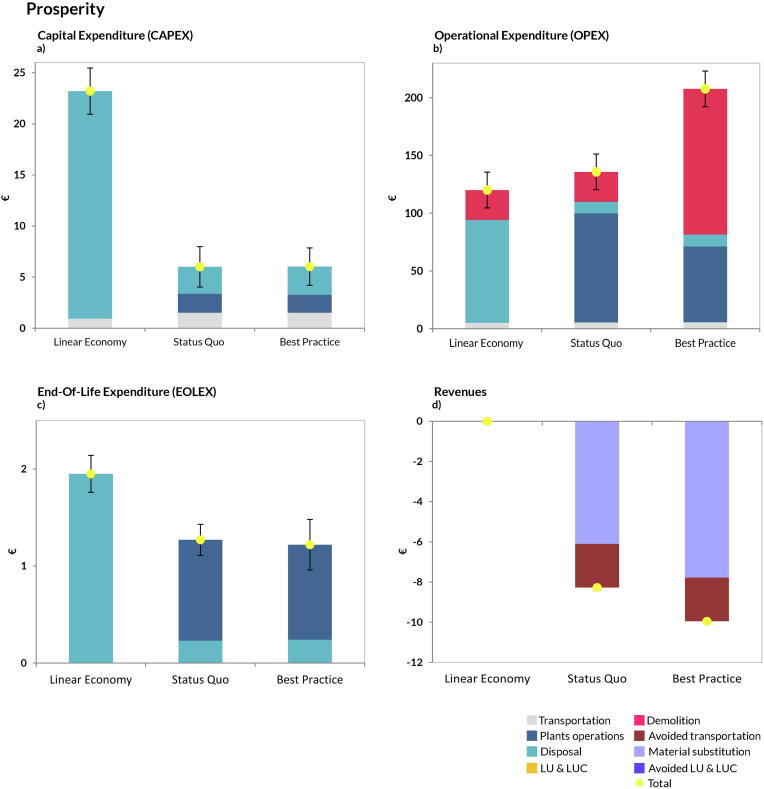
Disaggregated life-cycle results for the Area-of-Protection prosperity (FU: management of 1 t CDW). Positive values indicate burdens, while negative indicate savings. Plants operations: all activities involving material recycling (aggregates, wood, plastic, etc.); Material substitution: credits from replacement of virgin materials; Transportation: impacts related to transport of CDW flow to the management facilities and transport of RAs to the construction sites; Avoided transportation: saving related to avoided NAs transportation from quarry sites to plant facilities; LUCs induced: impacts due to land occupation, land transformation and land use changes (both direct and indirect); LUCs avoided: avoided land occupation, transformation and land use changes due to the reduction of mining activities from quarries; Disposal: all the impacts incurred by landfilling operations; Demolition: impacts coming from the process of demolition (both traditional and selective). Total represents the sum of all contributions. The uncertainty bar represents plus minus one standard deviation.

In the AoP Prosperity (Fig. 7) we observed a trade-off (OPEX; Fig. 7b) relative to the previous results: the scenario *Best Practice* incurred the highest OPEX due to the increased costs for selective demolition and recycling operations. As far as CAPEX is concerned, the *Best Practice* and the *Status Quo* scenarios had a comparable cost, but both were cheaper than the *Linear Economy* scenario (Fig. 6a). The *Best Practice* also achieved the highest revenues (Fig. 7d), while EOLEX were comparable to the S*tatus Quo* and lower than the *Linear Economy* (Fig. 7c). Comparable uncertainty ranges were observed across all scenarios and categories; rankings were not affected by the uncertainties.

### 4.4 Aggregation: Societal Life Cycle Costing & Multi-Criteria Decision Analysis

**Fig. 8:**
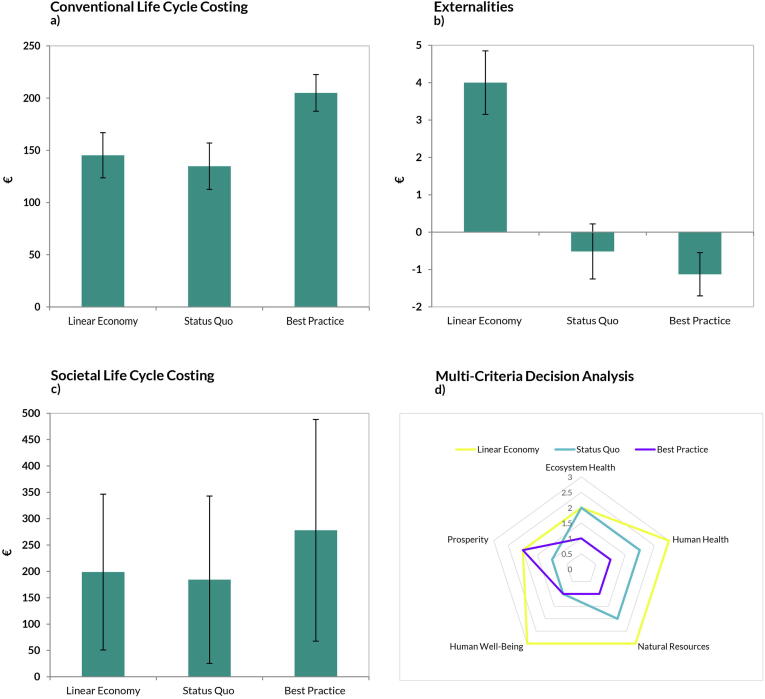
Illustration of the results for a) CLCC, b) Externalities c) SLCC, d) MCDA. The uncertainty bar represents plus minus one standard deviation.

Applying the MCDA technique proposed in Taelman et al. (2020) we obtained that *Best Practice* ranked first under all AoPs, except for Prosperity due to the increased operational costs (Fig. 8d). Under Prosperity, the *Status Quo* scored best. The *Linear Economy* scenario was the worst in 3 out of 5 AoPs (Human Health, Natural Resources and Human Well-Being) and scored comparable to the *Status Quo* in Ecosystem Health. (Fig. 8d). In terms of conventional costs (CLCC), we observe an increased CDW management cost moving from linear to more circular scenarios (Fig. 8a). The total CLCC was 134 €/t for Status Quo and 205 €/t for the scenario *Best Practice*. The increase was mainly due to higher recycling costs and especially higher costs for selective demolition, compared with the costs incurred with traditional demolition, moving from 26 €/t to 126 €/t. A similar pattern can be observed in the SLCC results, as the contribution of the external costs to the total societal cost was limited (around –1 to + 4 €/t).

## 5 Learnings and recommendations

### 5.1 Potential benefits of selective demolition and policy instruments needed

In EU, selective demolition practices, even if not widespread yet, could help increasing the waste quality and purity, enhancing the CDW diversion from landfill and reducing further land use consumption. Land is becoming an ever-increasing scarce resource (Munafò, 2019) and the avoided landfilling of demolition waste represents a very important environmental benefit (Blengini, 2009), alongside the reduced quarrying activity. Currently, the inert fraction represents a significant portion of CDW in many European Member States. It includes concrete, stones, bricks, tiles and ceramics and represents 20–70% of the total CDW flow (Deloitte, 2017). The systematic application of selective demolition can help increasing the breakdown of this fraction in well-separated material-specific streams that can be used to boost the production of high-quality RAs. Selective demolition would also help increasing the recycling of plastic, insulation (e.g. EPS or PUR) and wood. On top, metals are already usually separated and recycled. To facilitate the implementation of selective demolition, it is essential to start from the building construction phase, encouraging design for disassembly (Cristiano et al., 2021). Furthermore, for the purpose of overcoming the difficulty linked to the data collection phase, which saw us forced to interview the RAs producers, it would be appropriate to allow greater data transparency and traceability not only in the CDW generation and management phases, but also in the RAs production and final destination (Cristiano et al., 2021).

Our study shows that, while the potential environmental benefits of selective demolition are significant, the high economic costs may hinder its application and the resulting development of more circular economy actions in the construction sector. In view of this, a mixed combination of different tools may be envisaged to support a development in such direction, especially in terms of new policies, regulations and economic incentives and subsidies that might stimulate the development of a strong and competitive secondary-materials market for RAs (Wahlström et al., 2020). The first solution could be represented by introducing specific recycling targets for selective construction materials in place of those on the overall mass recycled, which may favour low quality recycling (e.g. of low-quality mixed aggregates for backfilling or environmental restoration projects). Considering the setting of appropriate material-based recycling rates to be reached by a certain year (e.g. 2030) and preparing for re-use and recycling targets for CDW (and its material-specific fractions) are already envisioned by Article 11(6) of the Waste Framework Directive (Directive 2008/98/EC as modified by Directive (EU) 2018/851). According to the global material stock and flow model for residential and service sector buildings towards 2050 developed by Deetman et al. (2020), the demand for construction materials seems continuing to globally increase (with estimated global projections of e.g. 31% for steel and 14% for cement compared to today according to their model) and closing the loop with high quality recycled materials appears unrealistic before the year 2050. Furthermore, Deetman et al. (2020) pointed out the important role played by the service sector buildings, because they may use certain materials at higher material intensity and they have a shorter average lifetime than residential buildings. Targeting these considerations to Europe appears paramount. CDW represents 25–30% of total waste volumes in Europe with a quite constant generation since 2010 (European Commission, 2019a, Enkvist and Klevnäs, 2018, Wahlström, 2020). Anyhow, reliability of statistics on CDW varies among Member States and some improvements appear needed (Deloitte, 2017, Wahlström, 2020). Despite most Member States have already met the recycle/recovery minimum target of 70% of non-hazardous CDW by 2020, recycled CDW is often employed in low quality applications or is downcycled. Higher-quality applications need to be fostered (European Commission (2020a), Wahlström, 2020), also by considering additional measures such as setting (the above-mentioned) targets for CDW material-specific fractions. To analyse the potential for these fraction recycling and to build robust forecasts, a study is currently ongoing on the European building stock. Indeed, according to Enkvist and Klevnäs (2018) and to Rose and Stegemann (2018), about 1% of EU buildings are renovated each year and new buildings are added (0.5–1.5% of the total stock) and significant renovations are expected in a close future, considering that about 45% of buildings are older than 50 years (Enkvist and Klevnäs, 2018). Building stock can be thus considered a major source of material-specific fractions and then if properly managed, can contribute significantly to the objectives of the Circular Economy Action Plan.

Another important instrument to foster increased and better waste recycling is the inclusion of recycled content criteria, particularly for RAs, in Green Public Procurements, both as technical specifications or award criteria. Furthermore, a regional sustainability certification for RAs can be promoted and higher disposal taxes could be inserted. Increasing landfilling charges and penalties for illegal mining activities and limestone sales could indeed foster the higher development of RAs (Cristiano et al., 2021).

To be accepted and used at the construction site, RAs need to comply with specific technical and environmental requirements. Currently, different factors hinder a more widespread use of RAs, such as the distrust of some construction companies against recycled materials due to their origin from waste (Wahlström, 2020) or the lack of knowledge on their technical performances, but also the low cost and the great availability of virgin materials at local level. For example, the quarrying activity in the Campania Region is intense and characterized by an ever-increasing number of extraction sites (Legambiente, 2017). It would, therefore, be necessary to encourage the use of RAs by making operational some regulatory instruments that require the use of a minimum amount of recycled content in public works (e.g. in Italy, the DM 203/2003 requires a minimum recycled content of 30%). It is also important to share information on the technical performances of RAs to improve the general awareness (Borghi et al., 2018), involving stakeholders in the development standards for secondary raw materials, enhancing trust and credibility (European Environment Agency, 2019). In this sense, RAs can complement NAs in a sustainable supply mix for the construction industry, i.e. a procurement from multiple sources, according to criteria of economic, environmental and social efficiency (Garbarino and Blengini, 2013). Enhancing the direct involvement of NAs production facilities in the RAs production chain could be an added value to promote the higher diffusion of RAs on the market, avoiding competition (Cristiano et al., 2021). Moreover, according to the Regional Plan for mining activities (2006), the number of quarries needs to be progressively reduced, also considering the deterioration and the biodiversity and land use losses at local level, going towards the restoration and re-naturalisation of exhausted quarries. Lastly, landscape disamenities and land use changes could be considered to correct the market for the negative externalities incurred by these activities.

### 5.2 Advances and limitations of the study

Striving to fill the gaps emerged with the initial literature review, related to assessment of costing, land use, and selective demolition impacts, we assessed the economic, social, and environmental pillars linked to the management of CDW in a selected Italian Region (Campania) considering the priorities of local stakeholders. This sustainability assessment of CDW management is the first of its kind, as previous studies focused on the environmental dimension (Mercante et al., 2012, Li et al., 2020) or on the economic costs (Di Maria et al., 2020). Similarly, to the proposed study, Di Maria et al. (2018) combine LCA and LCC to analyse the environmental and economic impacts of four alternative CDW management scenarios, even if the social component is missing. The environmental result of their study is in line with our results, since landfilling shows the highest environmental impacts, while incurring higher costs relative to our results because of the more expensive landfill tax in the area. Likewise, the results provided by Borghi et al. (2018) demonstrate the environmental advantages of landfill avoidance, but the economic and social perspectives are left behind. Jain et al. (2020) showcase that integrated wet recycling is environmentally sound alternative compared to landfilling in most of the cases, while Rondinel-Oviedo (2021), after performing some surveys and interviews to the main stakeholders, underline the importance to provide incentives for selective demolition.

Highlighting the trade-offs between AoPs, notably costs and environment, alongside including uncertainty propagation, we produced comprehensive information for policy- and decision-makers as envisioned in the EU impact assessment guidelines for better regulations (European Commission, 2017). In the effort to convey a clear message to local stakeholder and decision-makers, we performed a final aggregation applying MCDA and SLCC. While the former proposes a relative ranking of the scenarios assessed per each AoP, without compensating between the three pillars (e.g. social and/or economic costs with environmental impacts), the SLCC instead obtains an aggregated score as sum of budget and external costs (expressed as shadow prices) which implies an inevitable compensation across social, economic, and environmental pillars. It should be observed that the results obtained with the two methods are different because the results of the SLCC in this study are strongly driven by the conventional costs while externalities have minor impact on the final magnitude. Moreover, we also focused on land use-related impacts that are rarely considered (the only example we found is the study by Bidstrup et al., 2015). We highlight that including such impacts is particularly important for the impact categories of Land Use and Urban Space Consumption.

The limitations of the study are related to: i) data on CDW composition as obtained after selective demolition, ii) external costs for LUCs, and iii) quality and expected use of RAs. With respect to the first, current available data on CDW composition, i.e. as available from local agencies (ARPA) and scientific-technical studies, are the result of the application of traditional demolition practices. This means that the actual content of selected inert fractions (e.g. insulation, wood, gypsum, and concrete and stones), usually ranging between 20 and 70% (Deloitte, 2017), may be underestimated as a sizeable portion of the waste is typically classified as ‘mixed CDW’. For our purposes, specific data did not exist, and we had to take assumptions based on literature and best-guess estimates; we see therefore an urgent need for in-depth studies to improve the definition of the regional/local CDW composition. Such gap could be covered combining top-down building stock modelling (Metabolic, 2020) with waste characterisation studies *ex-post* selective demolition to derive a more detailed material breakdown. As for the second limitation, to describe the impact of LUCs we applied the figures proposed in Martinez-Sanchez et al. (2016). As also discussed in that study, the value used for LUCs and external costs of disamenities may underestimate the actual external cost as this is highly affected by the general uncertainty linked to the evaluation methodologies as well as the variability of different studies. While we applied existing knowledge and available figures from the literature, in general we observe a lack of studies investigating on the external costs of LUCs. Lastly, we observe that poor data exist on the final quality of RA produced from the treatment of CDW. In our case, to obtain information on the quality produced at the regional scale we performed a survey asking the main stationary recycling plants in the Region to provide quantitative estimates on their products. While this exercise can be replicated and extended to other Regions, we believe that such information should be made widely available, also in the endeavour to promote a market for local circular practices.

## 6 Conclusions

This study assessed the sustainability of CDW management in the Campania Region, Italy, encompassing environmental, social, and economic dimensions. Three scenarios were investigated: the status quo, a scenario reflecting linear economy (for benchmarking) and a scenario reflecting the implementation of best practices, involving selective demolition and increased material recycling. Our findings indicate that implementing selective demolition and increasing recycling generate environmental and social benefits, but at the same time higher conventional costs overall. According to our findings, the major contribution to the costs was selective demolition that was significantly more expensive than the conventional (ca. 6.2 €/t against 1.2 €/t). However, we estimated that the CO2-eq. savings per tonne can increase up to 88% applying selective demolition and increased recycling, relative to the status quo. Bearing the higher costs in mind, tools such as Green Public Procurement and new legislative targets may help steering the market and fostering more circular management practices in the construction sector.

## Disclaimer

The views expressed are purely those of the authors and may not in any circumstances be regarded as stating an official position of the European Commission.

## Declaration of Competing Interest

The authors declare that they have no known competing financial interests or personal relationships that could have appeared to influence the work reported in this paper.
