# Supplementary material for: Sustainability assessment of Construction and Demolition Waste management applied to an Italian case
Source: Waste Manag. 2021 Jun 1;128:83–98. doi: 10.1016/j.wasman.2021.04.031 (PMC8183595; doi:10.1016/j.wasman.2021.04.031)
Supplement: Supplementary data 1 [file mmc1.docx]

**Supporting Information for the article:**

**Sustainability assessment of Construction and Demolition Waste management applied to the case of the Campania Region (Italy)**

Silvia Iodice^ac*^, Elena Garbarino^b^, Maria Cerreta^c^, Davide Tonini^b^

^a^European Commission, Joint Research Centre (JRC), Ispra, Italy

^b^European Commission, Joint Research Centre (JRC), Seville, Spain

^c^University of Naples Federico II, Department of Architecture

*Corresponding author, email: silvia.iodice@ec.europa.eu

**Table of Contents**

[1. Modelling the CDW composition 3](#_Toc52978787)

[2. Treatment pathways: partitioning matrix 5](#_Toc52978788)

[3. Survey respondents details 6](#_Toc52978789)

[4. Inventory data: Technologies and processes 7](#_Toc52978790)

[5. Inventory data: land use changes 10](#_Toc52978791)

[6. Inventory data: disamenities 11](#_Toc52978792)

[7. Inventory data: occupational health 14](#_Toc52978793)

[References 15](#_Toc52978794)

# **1. Modelling the CDW composition**

The composition of the CDW flow, identified through the European Waste Catalogue (EWC) code 17, is detailed below and has been aggregated according to the material fractions available in the EASETECH database (Table S1).

**Table S1a.** Material fractions composing the CDW as modelled in the study (% of wet weight basis): traditional demolition.

| **Material Fraction** | **Share (%)**  **Traditional demolition** | **EWC codes** |
| --- | --- | --- |
| **Stones and concrete** | 4.84 | 170101: concrete |
|  |  | 170102: bricks |
|  |  | 170107: mixtures of concrete, bricks, tiles and ceramics other than those mentioned in 170106 |
| **Soil** | 34.85 | 170504: soil and stones other than those mentioned in 170503 |
|  |  | 170506: dredging spoil other than those mentioned in 170505 |
|  |  | 170508: track ballast other than those mentioned in 170507 |
| **Aluminium** | 0.58 | 170402: aluminium |
| **Steel** | 11.15 | 170405: iron and steel |
| **Other metals** | 1.51 | 170401: copper bronze brass |
|  |  | 170403: lead |
|  |  | 170404: zinc |
|  |  | 170407: mixed metals |
|  |  | 1704011: cables other than those mentioned in 170410 |
| **Ceramics** | 0.05 | 170103: tiles and ceramics |
| **Wood** | 0.2 | 170201: wood |
| **Glass** | 0.03 | 170202: glass |
| **Plastic** | 0.01 | 170203: plastic |
| **Bituminous mixtures** | 01.24 | 170302: bituminous mixtures other than those mentioned in 170301 |
| **Insulation materials** | 0.06 | 1700604: insulation materials other than those mentioned in 170601 and 170603 |
| **Gypsum** | 0.14 | 70802: gypsum-based construction materials other than those mentioned in 170801 |
| **Mixed CDW** | 36.34 | 170904: mixed construction and demolition wastes other than those mentioned in 170901, 170902 and 170903 |

**Table S1b.** Material fractions composing the CDW as modelled in the study (% of wet weight basis): selective demolition

| **Material Fraction** | **Share (%)**  **Selective demolition** | **EWC codes** |
| --- | --- | --- |
| **Stones and concrete** | 53.44 | 170101: concrete |
|  |  | 170102: bricks |
|  |  | 170107: mixtures of concrete, bricks, tiles and ceramics other than those mentioned in 170106 |
| **Soil** | 14.85 | 170504: soil and stones other than those mentioned in 170503 |
|  |  | 170506: dredging spoil other than those mentioned in 170505 |
|  |  | 170508: track ballast other than those mentioned in 170507 |
| **Aluminium** | 0.58* | 170402: aluminium |
| **Steel** | 11.15* | 170405: iron and steel |
| **Other metals** | 1.51* | 170401: copper bronze brass |
|  |  | 170403: lead |
|  |  | 170404: zinc |
|  |  | 170407: mixed metals |
|  |  | 1704011: cables other than those mentioned in 170410 |
| **Ceramics** | 0.05 | 170103: tiles and ceramics |
| **Wood** | 6.10 | 170201: wood |
| **Glass** | 0.41 | 170202: glass |
| **Plastic** | 0.01 | 170203: plastic |
| **Bituminous mixtures** | 01.24 | 170302: bituminous mixtures other than those mentioned in 170301 |
| **Insulation materials** | 1.21 | 1700604: insulation materials other than those mentioned in 170601 and 170603 |
| **Gypsum** | 0.14 | 70802: gypsum-based construction materials other than those mentioned in 170801 |
| **Mixed CDW** | 0 | 170904: mixed construction and demolition wastes other than those mentioned in 170901, 170902 and 170903 |

*We did not increase the collected amount of metals in selective demolition because we exclude metals from our boundaries

# **2. Treatment pathways: partitioning matrix**

**Table S2.** Mass partitioning of the CDW material fractions to the different treatment routes as modelled in the study (% of wet weight collected)

|  | **Stationary Recycling** | **Mobile Recycling** | **Recycling** | **Chemical Physical Biological Plant** | **Landfill *** |
| --- | --- | --- | --- | --- | --- |
| **Bituminous mixture** | 48.7% | 43.2% |  | 0.1% | 8.1% |
| **Glass** |  |  | 64.9% | 0.64% | 34.4% |
| **Mixed CDW** | 47.5% | 42.1% |  | 0.19% | 10.2% |
| **Insulation materials** |  |  | 29.8% | 5.23% | 64.9% |
| **Soil** | 46.5% | 41.2% |  | 0.58% | 11.7% |
| **Aluminium** |  |  | 80.9% | 0.03% | 19% |
| **Steel** |  |  | 80.9% |  | 19% |
| **Other metals** |  |  | 36.3% | 0.02% | 13.6% |
| **Stones and concrete** | 49.4% | 43.8% |  | 0.02% | 6.7% |
| **Ceramics** | 33.7% | 29.9% |  | 0.07% | 36.3% |
| **Plastic** |  |  | 0.3% | 5.06% | 94.6% |
| **Wood** |  |  | 58.6% | 0.29% | 41.1% |
| **Gypsum** | 40% | 35.5% |  | 0.2% | 24.3% |

*Landfill comprises also the fractions sent to storage and temporary storage facilities, as these are intermediate steps prior to final landfilling.

# **3. Survey respondents details**

The following data come from MUD database, complemented with the data from the regional observatory on waste management in the Campania Region (ORGR)^[[1]](#footnote-1)^, as well as with telephone survey with the main stationary recycling plants located in the Region. Out of the 23 companies selected, 11 responded to the telephone interview.

**Table S3.** Details on stationary recycling plants from the survey

|  | **Annual treated quantity (t)** | **RAs qualities according to the survey** | | |
| --- | --- | --- | --- | --- |
|  |  | **RAs Tipe A (%)** | **RAs Tyoe B (%)** | **RAs Type C (%)** |
| Stationary plant 1 | 271,265 |  |  |  |
| Stationary plant 2 | 158,924 |  | 65 | 35 |
| Stationary plant 3 | 132,645 |  |  |  |
| Stationary plant 4 | 106,350 |  |  |  |
| Stationary plant 5 | 104,427 |  |  |  |
| Stationary plant 6 | 91,000 |  |  |  |
| Stationary plant 7 | 89,365 | 10 | 90 |  |
| Stationary plant 8 | 78,724 |  |  |  |
| Stationary plant 9 | 65,074 |  |  |  |
| Stationary plant 10 | 58,895 | 30 |  | 70 |
| Stationary plant 11 | 55,098 |  |  |  |
| Stationary plant 12 | 52,463 |  |  |  |
| Stationary plant 13 | 48,000 |  | 100 |  |
| Stationary plant 14 | 40,852 |  |  |  |
| Stationary plant 15 | 31,388 | 20-30 | 20-30 | 20-30 |
| Stationary plant 16 | 28,107 |  |  |  |
| Stationary plant 17 | 27,702 | 20 | 70 | 10 |
| Stationary plant 18 | 19,500 |  | 100 |  |
| Stationary plant 19 | 16,199 |  | 100 |  |
| Stationary plant 20 | 15,381 | 25 | 25 | 50 |
| Stationary plant 21 | 6,201 |  |  |  |
| Stationary plant 22 | 4,987 | 20 | 60 | 20 |
| Stationary plant 23 | 2,113 |  | 100 |  |
| **Total** | **1,504,660** |  |  |  |

# **4. Inventory data: Technologies and processes**

**Table S4a.** Inventory data used to model the CDW treatment and management costs: stationary recycling plant

| **Stationary Recycling Plant** | **Description** | **Unit** | **Default Value** | **Sources** | **Range assumed** |
| --- | --- | --- | --- | --- | --- |
|  | **Technical Input Data** | | | | |
|  | Electricity | MJ/t | 3.605 | Blengini and Garbarino, 2010 | 4.326 – 2.884 |
|  | Diesel | l/t | 0.680 | Blengini and Garbarino, 2010 | 0.816 – 0.544 |
|  | Transport distance (CDW to treatment plant) | km*kg | 45 | Calculated by the authors according to plants localization | 30 – 700 |
|  | Transport distance (NAs type A from extraction site to construction site) | km*kg | 50 | Blengini and Garbarino, 2010 | 30 – 70 |
|  | Transport distance (NAs type B from extraction site to construction site) | km*kg | 25 | Blengini and Garbarino, 2010 | 20 – 30 |
|  | Transport distance (NAs type C from extraction site to construction site) | km*kg | 15 | Blengini and Garbarino, 2010 | 10 – 20 |
|  | **Technical Output Data** | | | | |
|  | RAs type A | % | 16.6 | Telephone interviews |  |
|  | RAs type B | % | 59 | Telephone interviews |  |
|  | RAs type C | % | 24.4 | Telephone interviews |  |
|  | Transport distance (RAs type A treatment plant to construction site) | km*kg | 35 | Blengini and Garbarino, 2010 | 30 - 40 |
|  | Transport distance (RAs type B treatment plant to construction site) | km*kg | 17,5 | Blengini and Garbarino, 2010 | 15 – 20 |
|  | Transport distance (RAs type C treatment plant to construction site) | km*kg | 10,5 | Blengini and Garbarino, 2010 | 7 – 14 |
|  | **Economic Input Data** | | | | |
|  | Investment | €/year | 365,104 | Garbarino and Blengini, 2013 | 228,691 – 525,589 |
|  | Land acquisition | €/m^2^ | 70 | Market research | 2 – 150 |
|  | Annual maintenance | €/year | 182,000 | Garbarino and Blengini, 2013 | 114,000 – 262,000 |
|  | Annual insurance | €/year | 22,750 | Garbarino and Blengini, 2013 | 14,250 – 32,750 |
|  | Labour | €/year | 368,000 | Garbarino and Blengini, 2013 | 368,000 – 736,000 |
|  | End of life | €/year | 229,541 | Home and Communities Agency, 2015 | 183,633 – 275,449 |
|  | Diesel | €/l | 0.432 |  | 0.346 – 0.518 |
|  | Electricity | €/kWh | 0.153 |  | 0.122 – 0.184 |
|  | **Economic Output Data** | | | | |
|  | RA type A cost | €/kg | 0.015 | Market research | 0.012 – 0.018 |
|  | RA type B cost | €/kg | 0.011 | Market research | 0.08 – 0.013 |
|  | RA type C cost | €/kg | 0.09 | Market research | 0.07 – 0.010 |

**Table S4b.** Inventory data used to model the CDW treatment and management costs: mobile recycling plant

| **Mobile Recycling Plant** | **Description** | **Unit** | **Default Value** | **Sources** | **Range assumed** |
| --- | --- | --- | --- | --- | --- |
|  | **Technical Input Data** | | | | |
|  | Diesel | l/t | 0.688 | Blengini and Garbarino, 2010 | 4.326 – 2.884 |
|  | Electricity | MJ/t | 3.605 | Blengini and Garbarino, 2010 | 0.816 – 0.544 |
|  |  |  |  |  |  |
|  | Transport distance (CDW to treatment plant) | Km*kg | 15 | Blengini and Garbarino, 2010* | 15 – 100 |
|  | Transport distance (NAs type C from extraction site to construction site) | Km*kg | 15 | Blengini and Garbarino, 2010 | 10 – 20 |
|  | **Technical Output Data** | | | | |
|  | RAs type C | % | 100 | Telephone interviews |  |
|  | Transport distance (RAs type C treatment plant to construction site) | Km*kg | 10,5 | Blengini and Garbarino, 2010 | 7 – 14 |
|  | **Economic Input Data** | | | | |
|  | Investment | €/year | 50,520 | Garbarino and Blengini, 2013 | 50,520 – 104,315 |
|  | Land rent | €/y | 6,540 | Market research | 3,600 – 7,848 |
|  | Annual maintenance | €/year | 25,183 | Garbarino and Blengini, 2013 | 25,583 – 52,000 |
|  | Annual insurance | €/year | 3,148 | Garbarino and Blengini, 2013 | 3,148 – 6,500 |
|  | Labour | €/year | 227,700 | Garbarino and Blengini, 2013 | 227,700 – 445,400 |
|  | Diesel | €/l | 0.432 |  | 0.346 – 0.518 |
|  | Electricity | €/kWh | 0.153 |  | 0.122 – 0.184 |
|  | **Economic Output Data** | | | | |
|  | RA type C cost | €/kg | 0.09 | Market research | 0.07 – 0.010 |

*Due to the impossibility to localize mobile plants, transport distance has been taken from literature

**Table S4c.** Inventory data used to model the CDW treatment and management costs: recycling plant

| **Recycling Plant** | **Description** | **Unit** | **Default Value** | **Sources** | **Range assumed** |
| --- | --- | --- | --- | --- | --- |
|  | **Technical Input Data** | | | | |
|  | Glass recycling |  |  | Ecoinvent centre, 2019 |  |
|  | Plastic recycling |  |  | Faraca et al., 2019 |  |
|  | Wood recycling |  |  | Faraca et al., 2019 |  |
|  | Insulation materials recycling |  |  |  |  |
|  | Transport distance (CDW to treatment plant) | km*kg | 106 | Calculated by the authors according to plants localization | 10 – 700 |
|  | **Economic Input Data** | | | | |
|  | Glass recycling | €/kg | 0.03 | https://resource-recycling.com/ | 0.03 – 0.045 |
|  | Plastic recycling | €/kg | 0.160 |  | 0.160 – 0.321 |
|  | Wood recycling | €/kg | 0.05 |  | 0.05 – 0.1 |
|  | Insulation materials recycling | €/kg | 0.160 |  | 0.160 – 0.321 |
|  | Labour | €/year | 227,700 |  | 227,700 – 445,400 |
|  | Diesel | €/l | 0.432 |  | 0.346 – 0.518 |
|  | Electricity | €/kWh | 0.153 |  | 0.122 – 0.184 |
|  | **Economic Output Data** | | | | |
|  | Recycled glass cost | €/kg | 0.045 | https://www.borsinorifiuti.com/2020/ | 0.036 – 0.054 |
|  | Recycled plastic cost | €/kg | 0.68 | https://www.borsinorifiuti.com/2020 | 0.68 – 1.36 |
|  | Recycled wood cost | €/kg | 0.05 | https://www.borsinorifiuti.com/2020 | 0.03 – 0.045 |
|  | Recycled polystyrene cost | €/kg | 0.62 | https://www.plasticfinder.it/vetrina/pub/POECNUP | 0.62 – 1.24 |

**Table S4d.** Inventory data used to model the CDW treatment and management costs: landfill plant

| **Landfill Plant** | **Description** | **Unit** | **Default Value** | **Sources** | **Range assumed** |
| --- | --- | --- | --- | --- | --- |
|  | **Technical Input Data** | | | | |
|  | Electricity | kWh/t | 1.14 | Blengini and Garbarino, 2010 |  |
|  | Diesel | l/t | 20.23 | Blengini and Garbarino, 2010 |  |
|  |  |  |  |  |  |
|  | Transport distance (CDW to treatment plant) | Km*kg | 210 | Calculated by the authors according to plants localization | 100 - 700 |
|  | **Economic Input Data** | | | | |
|  | Investment | €/year | 2,219,435 | Martinez-Sanchez et al., 2015 | 1,646,400 – 2,469,000 |
|  | Annual maintenance | €/year | 276,591 | Martinez-Sanchez et al., 2015 | 221,273 – 276,591 |
|  | Annual insurance | €/year | 138,295 | Martinez-Sanchez et al., 2015 | 110,636 – 138,295 |
|  | Analysis leachate | €/year | 132,990 | Martinez-Sanchez et al., 2015 | 106,392 – 159,588 |
|  | Analysis groundwater | €/year | 27,456 | Martinez-Sanchez et al., 2015 | 21,965 – 32,947 |
|  | Labour | €/year | 227,700 | Martinez-Sanchez et al., 2015 | 227,700 – 445,400 |
|  | End of life | €/year | 194,611 | Home and Communities Agency, 2015 | 155,689 – 233,533 |
|  | Diesel | €/l | 0.432 |  | 0.346 – 0.518 |
|  | Electricity | €/kWh | 0.153 |  | 0.122 – 0.184 |
|  | Landfill Tax | €/t | 8 | Garbarino and Blengini, 2013;  Market research |  |

**Table S4e.** Inventory data used to model traditional and selective demolition

| **Traditional demolition** | **Description** | **Unit** | **Default Value** | **Sources** | **Range assumed** |
| --- | --- | --- | --- | --- | --- |
|  | **Technical Input Data** | | | | |
|  | Electricity | kWh/t | 0.1 | Pantini and Rigamonti, 2020 |  |
|  | Diesel | l/t | 1.17 | Pantini and Rigamonti, 2020 |  |
|  | **Economic Input Data** | | | | |
|  | Labour | €/kg | 0.0012 | Coelho and De Brito, 2010 | 0.0010 – 0.0014 |
| **Selective demolition** | **Technical Input Data** | | | | |
|  | Electricity | kWh/t | 2.77 | Pantini and Rigamonti, 2020 |  |
|  | Diesel | l/t | 1.61 | Pantini and Rigamonti, 2020 |  |
|  | **Economic Input Data** | | | | |
|  | Labour | €/kg | 0.0062 | Coelho and De Brito, 2010 | 0.0050 – 0.0074 |

**Table S4F.** Inventory data used to model transport

| **Truck** | **Description** | **Unit** | **Default Value** | **Sources** | **Range assumed** |
| --- | --- | --- | --- | --- | --- |
|  | **Technical Input Data** | | | | |
|  | Diesel consumption | Kg/km | 0.00002 |  | 0.00002 – 0.00003 |
|  | **Economic Input Data** | | | | |
|  | Investment | €/year | 25,404 |  | 20,323 – 30,485 |
|  | Annual maintenance | €/year | 2,000 |  | 1,646 – 2,469 |
|  | Annual insurance | €/year | 16,270 |  | 13,016 – 19,524 |
|  | Labour | man*hour/kg waste | 0.00009 |  | 0.00007 – 0.00010 |

# **5. Inventory data: land use changes**

**Table S5a.** Inventory data used to model land use related to treatment facilities

| **Facility** | **Land occupied (m^2^)** | **Waste treated (kg/year)** | **m^2^*y/kg** | **Range assumed** |
| --- | --- | --- | --- | --- |
| **Stationary recycling plant** | 25,000 | 90,000,000 | 0.00028 | 0.000224 – 0.000336 |
| **Mobile recycling plant** | 10,000 | 90,000,000 | 0.000110 | 0.00088 – 0.000132 |
| **Recycling plant (average)** | 19,000 | 90,000,000 | 0.000210 | 0.000168 – 0.000252 |
| **Landfill** |  |  | 0.000080* | 0.000064 – 0.000096 |

Source: Blengini and Garbarino, 2010.

**Table S5b.** Inventory data used to model land use related to limestone quarries

|  | **Extracted quantity (t/y)** | **Surface (m^2^)** | **Transformation from** | **Transformation to** | **Occupation** | **Years of activity** |  |
| --- | --- | --- | --- | --- | --- | --- | --- |
| 1 | 4,380,000 | 260,000 | Arable land | Mineral extraction site | Mineral extraction site | 30 |  |
| 2 | 4,102,039 | 243,500 | Pasture | Mineral extraction site | Mineral extraction site | 45 |  |
| 3 | 86,266 | 51,439 | Arable land | Mineral extraction site | Mineral extraction site | 30 |  |
| 4 | 542,976 | 201,679 | Arable land | Mineral extraction site | Mineral extraction site | 30 |  |
| 5 | 916,608 | 341,016 | Arable land | Mineral extraction site | Mineral extraction site | 30 |  |
| 6 | 257,870 | 201,500 | Arable land | Mineral extraction site | Mineral extraction site | 30 |  |
| 7 | 124,854 | 74,319 | Arable land | Mineral extraction site | Mineral extraction site | 30 | **Range assumed** |
| **Total from arable** | | **6,308,574 t/y** | | **1,129,953 m^2^** | **1.79E-04 m^2^/t** | | **1.43E-04** –**2.15E-04** |
| **Total from pasture** | | **4,102,038 t/y** | | **243,500 m^2^** | **5.94E-05 m^2^/t** | | **4.7E-05** – **7.1E-05** |

**Table S5c.** Inventory data used to model greenhouse gas emissions associated to land use changes.

|  | **US** | **BR** | **EU28** | **US** | **BR** | **EU28** | **Total** | **PF** | **GHG** | **GHG** |
| --- | --- | --- | --- | --- | --- | --- | --- | --- | --- | --- |
| **Feedstock** | **%** | **%** | **%** | **NPP0** | **NPP0** | **NPP0** | **NPP0** | **Ratio** | **kg/(pw m2*y)** | **kg/(m2*y)** |
| quarry on EU arable | 0% | 0% | 100% | 0.58 | 0.86 | 0.59 | 0.59 | 1.04 | 0.013 | 0.01308803 |
| quarry on EU grassland | 0% | 0% | 100% | 0.58 | 0.86 | 0.59 | 0.59 | 1.04 | 0.063 | 0.06544014 |
| quarry on EU int. forest | 0% | 0% | 100% | 0.58 | 0.86 | 0.59 | 0.59 | 1.04 | 0.149 | 0.15477113 |
| quarry on EU ext. forest | 0% | 0% | 100% | 0.58 | 0.86 | 0.59 | 0.59 | 1.04 | 0.134 | 0.13919014 |

# **6. Inventory data: disamenities**

As many technologies distributed across the Country are aggregated in the model, many different plants can cause disamenities not only in the Campania Region, where the flow is produced and partially treated, but also in other Italian Regions where the rest of the flow is also managed.

Hence, for the disamenities calculation, the most representative stationary recycling plants and landfill plants were considered (Figure S1)


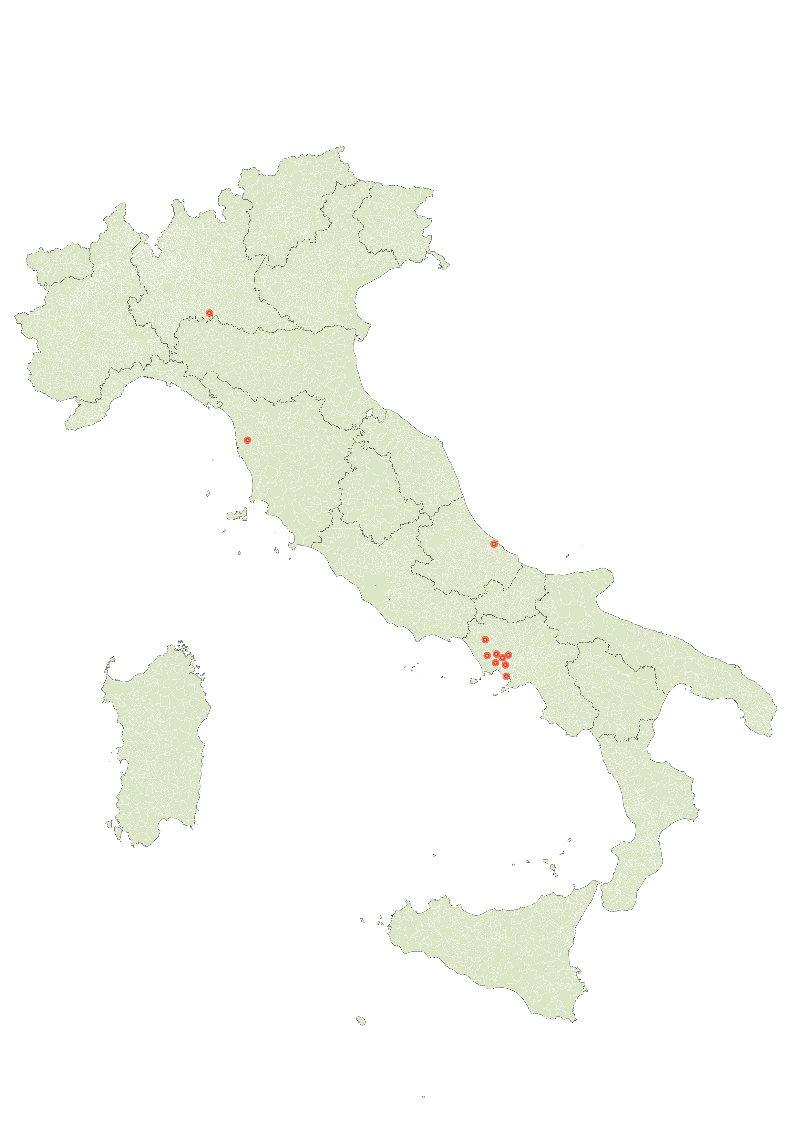


**Figure S1.** The most representative landfills and stationary recycling plants in the Country

Due to these aspects, it is not possible to apply a linear regression, as requested by the application of the Hedonic Price Method (Rosen, 1974), not knowing the characteristics of the properties surrounding the plants, therefore a simplified formula was applied. The most representative plants were spatially represented through Geographic Information System (GIS). Three different distance buffers were initially considered: 1 km, 3 km and 5 km (Figures S2, S2).

The different municipalities in the Campania Region are welded together. To this, it is added that these plants are medium-sized and do not affect house prices at certain distances. For these reasons, the 1 km buffer was chosen for the disamenities calculation, being both the 3 and 5 km buffers influenced by other aspects as well and not only by the plant presence.

There are three landfills for inert aggregates located outside the Campania Region. The 1 km buffer was also considered sufficient in this case for the calculation. Data concerning the number of houses coming from the National Institute of Statistics (ISTAT^[[2]](#footnote-2)^) where spatially represented in the GIS and crossed with the 1 km buffer, in order to evaluate the number of houses in a radius of 1 km from the considered plant (Table S6; S7). Details on the applied methodology are also available on Taelman et al. (2018).


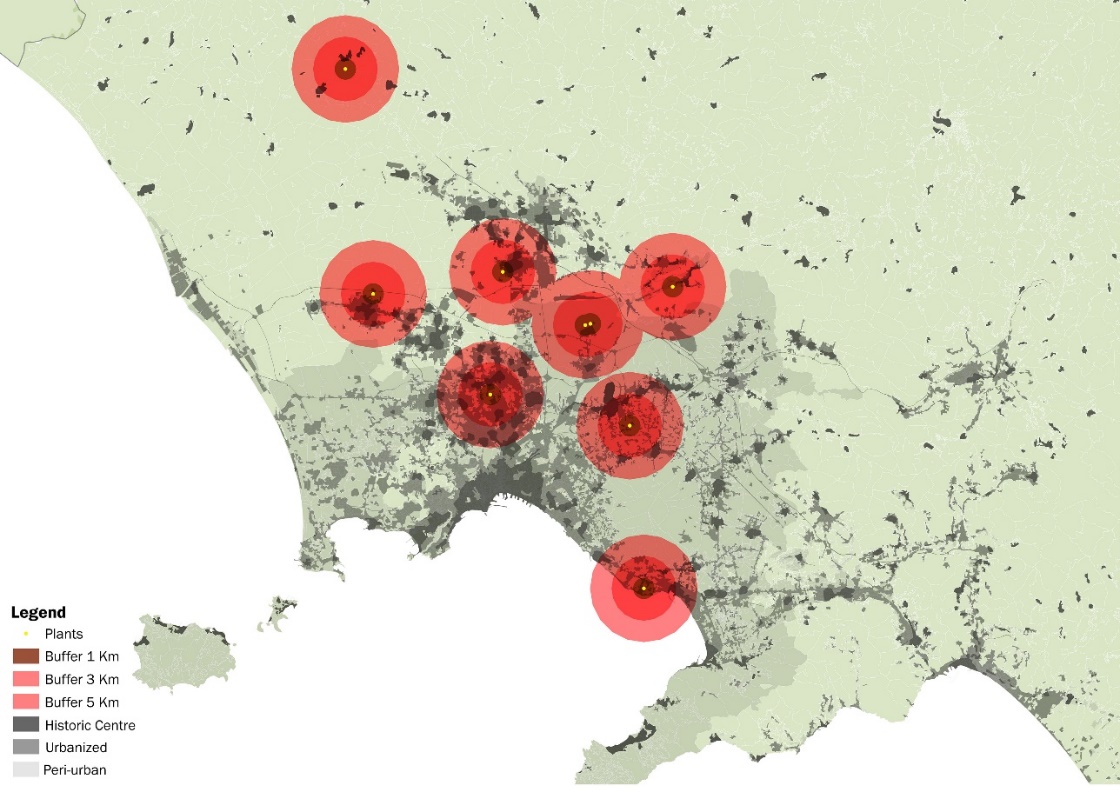


**Figure S2.** The analysed plants in the Campania Region and part of the Abruzzo Region with the 1-3-5-km buffers


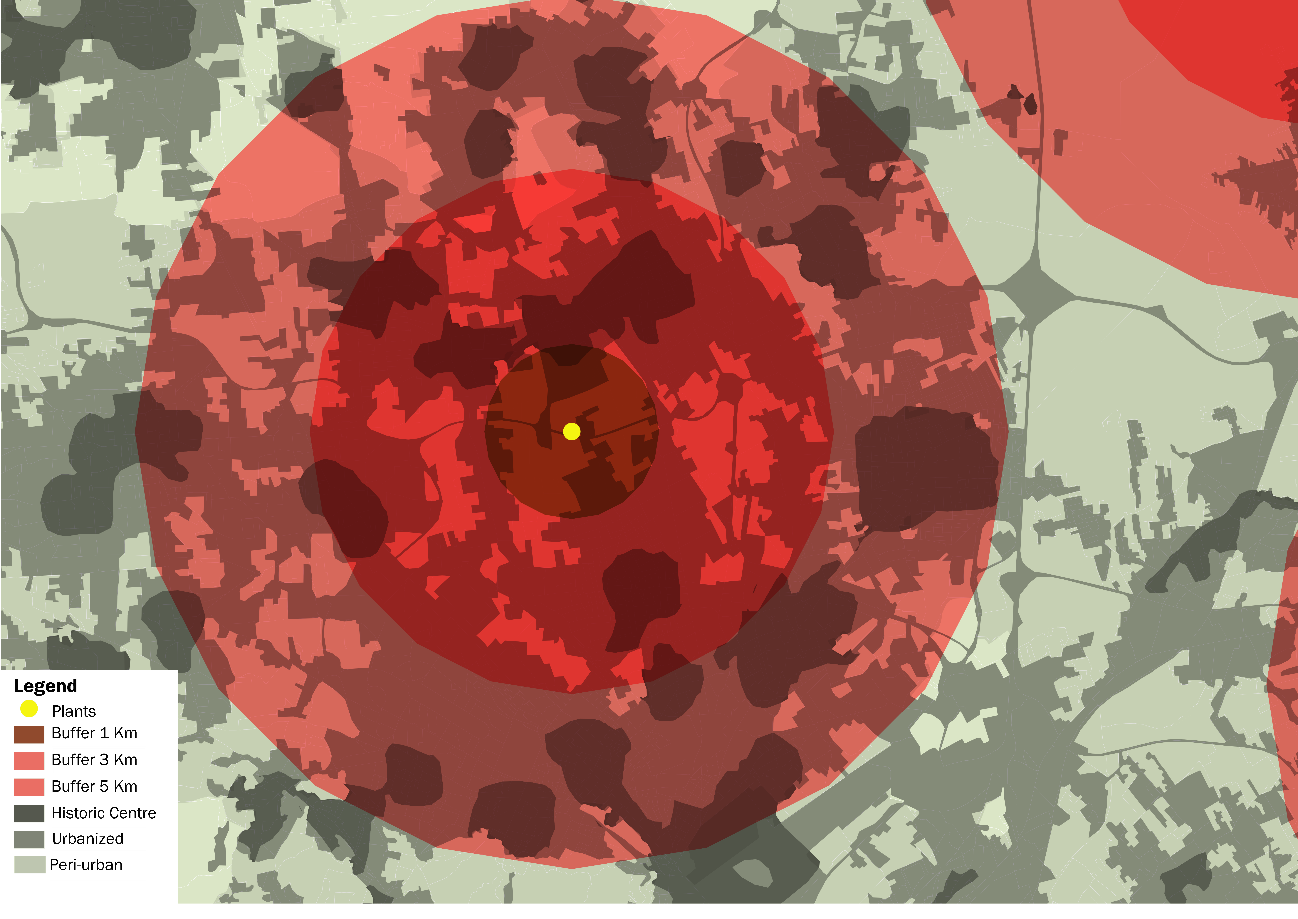


**Figure S3.** An example of stationary recycling plant

**Table S6a.** Disamenities calculation for landfills

| **Facility** | **Number of houses** | **Medium price area (€/m^2^)** | **Decrease (%)** | **Variation of property value (€/m^2^)** | **Average m^2^** | **Result (€)** |
| --- | --- | --- | --- | --- | --- | --- |
| 1 | 6 | 921 | 5% | 46,05 | 100 | 27,630 |
| 2 | 2978 | 884 | 5% | 44,2 | 100 | 13,162,670 |
| 3 | 3908 | 914 | 5% | 45,7 | 100 | 17,859,560 |
| 4 | 207 | 877 | 5% | 43,8 | 100 | 907,695 |
| 5 | 15 | 1314 | 5% | 65,7 | 100 | 98,550 |
| 6 | 59 | 813 | 5% | 40,65 | 100 | 239,835 |
| **Total** | **7173** |  | 5% |  |  | **32,296,030** |

| **Total usagerate (kg)** | **Years of activity** | **Total property price loss (€)** | **Disamenity** **(€/kg)** | **Range assumed** |
| --- | --- | --- | --- | --- |
| 1,574,239,000 | 25 | 32,296,030 | 0,02 | 0,016 – 0,024 |

**Table S6b.** Disamenities calculation for stationary recycling plants

| **Facility** | **Number of houses** | **Medium price area (€/m^2^)** | **Decrease (%)** | **Variation of property value (€/m^2^)** | **Average m^2^** | **Result (€)** |
| --- | --- | --- | --- | --- | --- | --- |
| 1 | 6 | 921 | 5% | 46,05 | 100 | 27,630 |
| 2 | 1316 | 1351 | 5% | 67,6 | 100 | 8,889,580 |
| 3 | 1064 | 1024 | 5% | 51,2 | 100 | 5,477,680 |
| 4 | 562 | 630 | 5% | 31,5 | 100 | 1,770,300 |
| 5 | 453 | 755 | 5% | 37,8 | 100 | 1,710,075 |
| 6 | 1745 | 566 | 5% | 28,3 | 100 | 4,938,350 |
| **Total** | **7173** |  | 5% |  |  | **22,783,350** |

| **Total usagerate (kg)** | **Years of activity** | **Total property price loss (€)** | **Disamenity** **(€/kg)** | **Range assumed** |
| --- | --- | --- | --- | --- |
| 3,166,030,000 | 25 | 22,783,350 | 0,007 | 0,0056 – 0,0084 |

As far as market values are concerned, the source is represented by the Italian real estate market^[[3]](#footnote-3)^, which represents a database of real estate quotes according to the municipalities and to the different areas within the same municipality (historic centre, suburbs, industrial zones, etc.). In order to calculate the change in the market value, the intermediate range between the most valuable location and the least valuable location was considered within a radius of 1 km with respect to the plant considered. As suggested by the simplified formula proposed by the European Commission (2014), the 5% decrease is applied to this intermediate price, verifying that this result is not equal to or lower than the less valuable location values, which will also be influenced by other features besides the presence of the plant.

# **7. Inventory data: occupational health**

**Table S7.** Occupational health

|  | **Number of accidents** | **Number of employees** | **Accidents/employees** | **Range assumed** |
| --- | --- | --- | --- | --- |
| **With means of transport involved** | 303^[[4]](#footnote-4)^ | 15163^[[5]](#footnote-5)^ | 0.830 | 0.664 – 0.996 |
| **Without means of transport involved** | 3136^4^ |  | 0.030 | 0.024 – 0.036 |

**References**

Blengini, G.A., Garbarino, E., 2010. Resources and waste management in Turin (Italy): the role of recycled aggregates in the sustainable supply mix. Journal of Cleaner Production 18, 1021–1030. <https://doi.org/10.1016/j.jclepro.2010.01.027>.

Garbarino, E., Blengini, G.A., 2013. The economics of construction and demolition waste (C&DW) management facilities, in: Handbook of Recycled Concrete and Demolition Waste. Elsevier, pp. 108–138. https://doi.org/10.1533/9780857096906.1.108

Coelho, A., De Brito, J., 2013. Conventional demolition versus deconstruction techniques in managing construction and demolition waste (CDW), in: Handbook of Recycled Concrete and Demolition Waste. Elsevier, pp. 141–185. https://doi.org/10.1533/9780857096906.2.141

European Commission (2014), ‘Guide to Cost-Benefit Analysis of Investment Projects: Economic Appraisal Tool for Cohesion Policy 2014-2020’, Publications Office of the European Union.

Faraca, G., Tonini, D., Astrup, T.F., 2019. Dynamic accounting of greenhouse gas emissions from cascading utilisation of wood waste. Science of The Total Environment 651, 2689–2700. https://doi.org/10.1016/j.scitotenv.2018.10.136

Home and Communities Agency, 2015. Guidance on dereliction, demolition and remediation costs. Available at: <https://assets.publishing.service.gov.uk/government/uploads/system/uploads/attachment_data/file/414378/HCA_Remediation_Cost_Guidance_2015.pdf> (accessed October 2020).

Martinez-Sanchez, V., Kromann, M. A. and Astrup, T. F. (2015) ‘Life cycle costing of waste management systems: Overview, calculation principles and case studies’, Waste Management. Elsevier Ltd, 36, pp. 343–355. doi: 10.1016/j.wasman.2014.10.033.

Rosen, S. (1974). Hedonic Prices and Implicit Markets: Product Differentiation in Pure Competition. Journal of Political Economy, 82(1), 34-55. Retrieved October 1, 2020, from http://www.jstor.org/stable/1830899

Taelman, S. E., Sanjuan, D., Tonini, D., Wandl, A., Dwulf, J. 2018. Deliverable 4.4: Definitive framework for sustainability assessment. Deliverable of the REPAiR Project, available at: http://h2020repair.eu/wp-content/uploads/2019/04/Deliverable-4.4-Definitive-framework-for-sustainability-assessment.pdf (accessed November 2020).

1. http://orr.regione.campania.it/ [↑](#footnote-ref-1)
2. https://www.istat.it/ [↑](#footnote-ref-2)
3. https://www.borsinoimmobiliare.it/ [↑](#footnote-ref-3)
4. https://dati.inail.it/opendata/default/Infortuni/index.html [↑](#footnote-ref-4)
5. http://dati.istat.it/ [↑](#footnote-ref-5)
